# Case report: Imaging of adrenal adenomatoid tumors: reports of two cases and review of literature

**DOI:** 10.3389/fonc.2024.1435143

**Published:** 2024-10-04

**Authors:** Yuanyuan Wu, Dongliang Hu, Manman Cui, Yan Liu, Xiuzhi Zhou, Duchang Zhai, Guohua Fan, Wu Cai

**Affiliations:** Department of Radiology, The Second Affiliated Hospital of Soochow University, Suzhou, ;China

**Keywords:** adenomatoid tumors, adrenal gland, computed tomography, magnetic resonance (MR), imaging

## Abstract

Adenomatoid tumors (ATs) are uncommon, benign tumors of mesothelial origin, most frequently found in the genital tracts of both sexes. Extragenital localization sites, such as adrenal glands, are extremely rare. Since patients with adrenal ATs have no obvious clinical symptoms, imaging examination has become important evidence for diagnosis. Although previous literature noted that the imaging findings of adrenal ATs were nonspecific, no relevant studies have confirmed this. We herein present two novel cases of adrenal ATs, confirmed by immunohistochemistry, and that were initially misdiagnosed as other, more common adrenal tumors based on clinical findings and preoperative imaging. Including our cases, we collected a total of 33 previously reported adrenal ATs and extracted all available imaging information from them, aiming to find some radiological characteristics of this rare tumor. Through the review, we identified some nonspecific imaging features of adrenal ATs; however, the final diagnosis still depends on pathology and immunohistochemistry results.

## Introduction

Adenomatoid tumors (ATs) are uncommon benign neoplasms of mesothelial origin, usually occurring in the genital tracts (1). Extragenital localization sites, such as adrenal glands, are extremely rare, and only fewer than 50 cases have been reported so far. Since patients with adrenal ATs usually present without clinical symptoms, imaging examinations have become an important part of evidence for diagnosis. However, previous reviews mainly focused on the pathology features, and no literature has revealed the imaging features of this rare tumor. Here, we report two new cases of adrenal ATs and review previously reported cases, with a focus on the imaging aspects of the tumors.

## Case presentation

### Timeline

#### Case 1

A 33-year-old man was admitted to the hospital because of an increased serum carbohydrate antigen 125 (CA125) level and an incidental adrenal mass observed on a computed tomography (CT) scan. He was asymptomatic, and further laboratory examinations revealed that urinary vanillylmandelic acid, serum cortisol, and ketosteroid levels were within normal limits. Adrenalectomy was performed under laparoscopy after an abdominal magnetic resonance (MR) imaging scan.

#### Case 2

A 28-year-old man presented to the hospital for a routine check-up, during which a mass lesion in the right adrenal gland was incidentally detected by ultrasonography. He reported no symptoms of palpitations, diaphoresis, flushing, or uncontrolled high blood pressure. After admission, further examinations such as CT and MR were performed, and the lesion, along with the affected adrenal gland, was excised.

### Imaging findings

**Figure 1:**
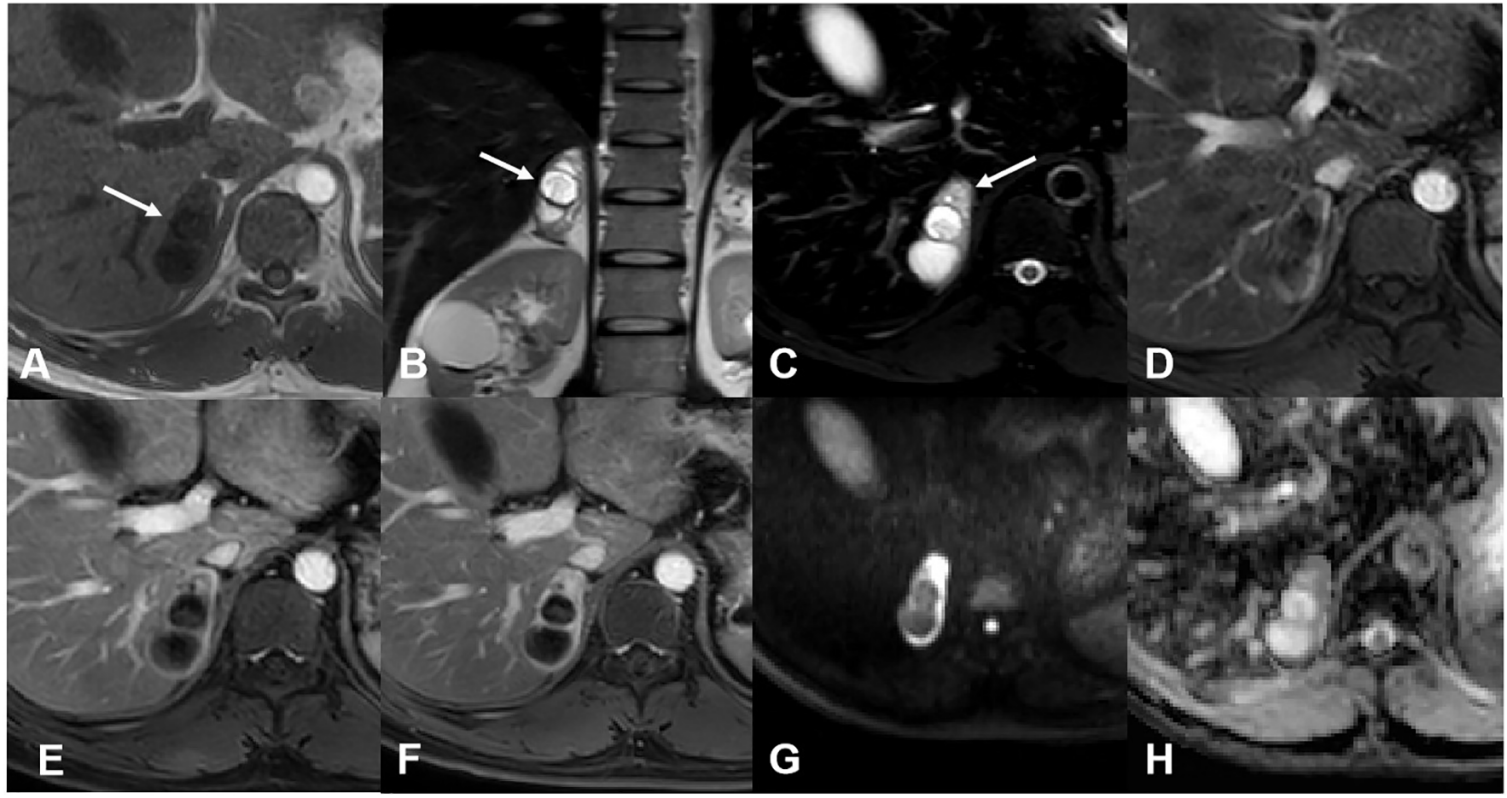
MR images of case 1. **(A)** The mass was hypointense on the T1-weighted image. **(B)** The T2-weighted image showed the mass was mainly cystic with some solid area in its more peripheral portion. **(C)** The solid area was hyperintense on the T2 SPAIR image (arrow). Enhanced images of arterial **(D)**, venous **(E)**, and delayed **(F)** phases showed gradual enhancement and delayed washout of the solid component. The cystic potion was not enhanced. Diffusion-weighted **(G)** and apparent dispersion coefficient **(H)** images showed no restricted diffusion of the solid component.

For case 1, MR images revealed a well-marginated, irregular mass measuring 5.2 cm × 2.4 cm in the right adrenal gland. The mass was mainly cystic, with a solid area in its more peripheral portion. The solid part was hyperintense on axial spectral attenuated inversion recovery (SPAIR) images and presented heterogeneous, marked enhancement after contrast administration, delineating the cystic area. There was no obvious restricted diffusion of the solid components (**Figure 1**). Based on the above findings, the radiologist suggested a schwannoma or a pheochromocytoma.

**Figure 2:**
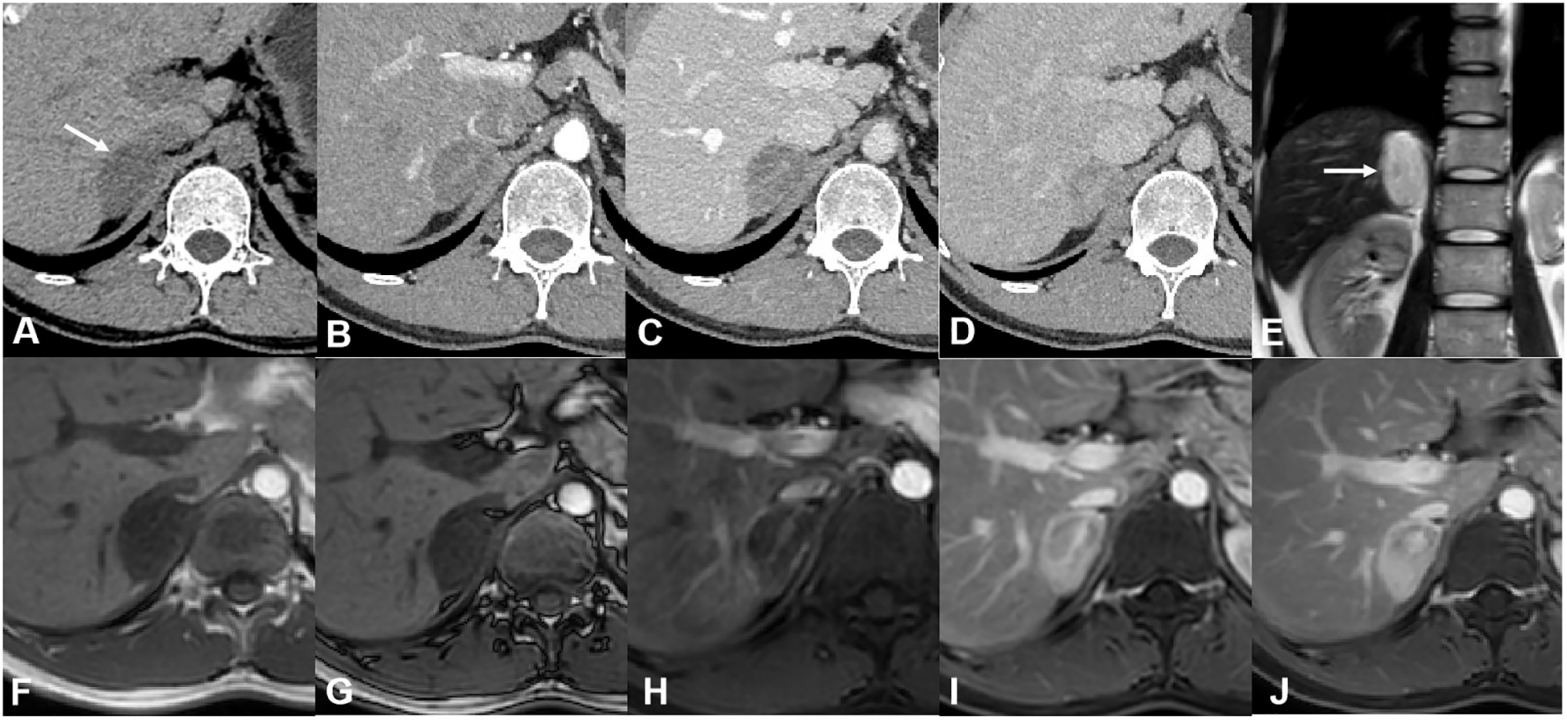
CT and MR images of case 2. **(A)** Axial plain CT images showed a well-defined mass with a mean attenuation value of 34 HU on the right adrenal region. Axial enhanced CT of arterial **(B)**, venous **(C)**, and delayed **(D)** phases showed moderate enhancement and delayed washout of the tumor (the mean attenuation value was 71 on the delayed phase). **(E)** Coronal T2-weighted image showed hyperintensity of the tumor. No signal change was shown between in-phase **(F)** and out-of-phase **(G)** axial T1-weighted images. **(H)** Enhanced MR images of the arterial phase showed a small vessel crossing the tumor. Venous **(I)** and delayed **(J)** phases of enhanced MR images showed the contrast agent progressively impregnating the tumor from the periphery to the center. There was a relatively poor enhancement area in the interior of the tumor.

For case 2, CT revealed a mixed-density lesion with well-defined margins in the right adrenal region, approximately 3.5 cm × 2.5 cm in size. The mass exhibited moderate enhancement with delayed washout, showing a mean attenuation value of 34 HU on plain CT and 71 HU on the delayed phase (in the range of moderate enhancement). On MR imaging, the lesion appeared as a mainly solid mass, hypointense on T1-weighted images and hyperintense on T2-weighted images. After contrast injection, the lesion was progressively impregnated by the contrast agent from the periphery to the center and presented delayed washout. Some interior areas of the tumor ultimately showed relatively poor enhancement (**Figure 2**). Based on these findings, a ganglioneuroma was presumed.

For both cases, no signal change was observed between in-phase and out-of-phase axial T1-weighted images. There were no suspicious hemorrhage or calcification foci, and no evidence of direct extension into surrounding structures or regional lymph node metastasis was found. No signs of recurrence or metastatic lesions were shown after 43 and 22 months of follow-up, respectively.

### Pathological findings

**Figure 3:**
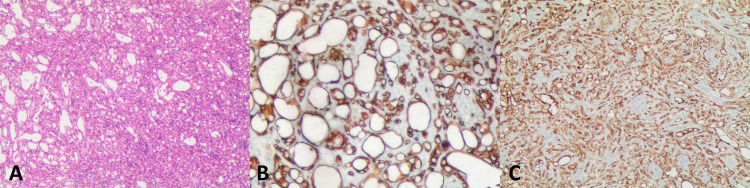
Histological features and immunohistochemistry associated with adrenal adenomatoid tumor. **(A)** The angiomatoid pattern of the tumor, which is composed of anastomosing, variably sized tubules lined by flattened or cuboidal cells (case 2, hematoxylin and eosin: × 15). **(B)** The tumor cells were diffusely positive for CK (× 400). **(C)** Tumor cells positive for calretinin (× 100). CK, cytokeratin.

On gross examination, the resected tumor of case 1 measured 4 cm × 2.5 cm × 1 cm, with multiple cystic foci inside, the largest of which was 2.5 cm in diameter. The tumor excised from the case 2 patient was 4 cm × cm 3.5 cm × 2.5 cm in size and had a solid, grayish-yellow cut surface. Microscopic analysis showed many cystic spaces (case 1) and anastomosing tubules (case 2) lined by flat or plump epithelioid cells. Some tumor cells contained intracytoplasmic vacuoles and eccentrically displaced nuclei, forming a signet ring cell appearance. All tumor cells had a low nuclear/cytoplasmic ratio, with no significant mitotic activity or nuclear pleomorphism. Tumor cells of the two cases showed strong immunoreactivity for AE1/AE3, calretinin, cytokeratin 7 (CK7), podoplanin (D2-40), and weak reactivity for Wilm’s tumor gene-1 (WT1), but were negative to chromogranin A (CgA), cluster of differentiation 34 (CD34), and synaptophysin (Syn). Based on these findings, adrenal ATs were diagnosed (**Figure 3**).

## Literature review

A total of 51 records from 1997 to 2024 were searched on PubMed using the keywords “adenomatoid tumor” and “adrenal gland”, and only the literature written in English was reviewed. Cases with severely incomplete information were not included. Ultimately, including our two cases, we listed a total of 33 patients with pathologically confirmed adrenal adenomatoid tumors and summarized their demographic information, imaging patterns and features, preoperative diagnoses, histopathological results, and follow-up times (2–25). Details can be found in the **Supplementary Material**. Among them, only 13 cases have relatively comprehensive imaging descriptions, which are further summarized in **Table 1**. To the best of our knowledge, this is the first time that imaging features of adrenal adenomatoid tumors have been aggregated and compared together with histological features.

**Table 1:** Radiological features of adenomatoid tumors of the adrenal gland.

Component of the tumor on imaging	Number of cases	Description of the tumor performance on unenhanced CT/MR in the article	Description of the tumor performance after enhancement	Component of the tumor on gross examination
Solid	1	Solid (15)	–	Solid and cystic
Solid and cystic (mainly solid)	3	Peripheral cystic Homogeneous, isointense to the spleen (solid) (3)	Marked enhancement (solid)	Solid and cystic
		Heterogeneous (solid), hypodense (cystic), and intermediate density zone (23)	–	Solid
		Mixed density Hypointense on T1 and hyperintense on T2 (solid) (our case 2)	Moderate enhancement Impregnated progressively from the periphery to the center Delayed washout	Solid
Solid and cystic (mainly cystic)	1	Peripheral solid Hyperintense on SPAIR (solid) (our case 1)	Heterogeneous marked enhancement (solid)	Solid and cystic
Cystic	3	A cyst of hepatic, renal, or adrenal origin (8)	–	Cystic
		A giant cystic of the liver (13)	–	Cystic
		Polycystic Mixed density Uneven thickness of the cyst wall (25)	Mild enhancement	Cystic
Not mentioned solid/cystic	5	Heterogeneous (7, 17)	Peripheral enhancement Hypodense in interior (7)	Solid (7, 17)
		Hypodense (19)	Slight peripheral enhancement	Cystic
		Uneven density (18)	Majority unenhancement Mild to moderate enhancement of a small part	Solid
		Mostly hyperintense, internal hypointense (nodular and thin septal components) on STIR (21)	Enhancement of internal components	Solid and cystic

### Demographic and clinical findings

Patient’s ages at diagnosis ranged from 22 to 64 years (mean, 38 years; median, 35 years), with 31 cases occurring in men and only 2 in women. Contrary to the previously reported prevalence in the left adrenal gland, we found that this tumor occurred more on the right side (21/33). All tumors presented in patients as incidental radiological, surgical, or autopsy findings. The tumor in more than 80% (27/33) of patients was discovered incidentally during radiological examinations, in three patients during autopsy, and in one patient during surgery for resection of rectal adenocarcinoma. Most of the patients were asymptomatic. Hypertension was found in seven cases of adrenal AT. Three patients had nephrolithiasis. Other symptoms included hematuria, palpitations and dizziness, syncope, and chronic abdominal pain. The tumors in other patients were found during investigations for acute cholecystitis, diverticulitis, family genetic diseases, acquired immune deficiency syndrome, and abnormal serum tumor markers. Based on the autopsy results, the causes of death for two of the three patients were identified as generalized respiratory failure resulting from disseminated Coccidiosis and acute coronary thrombosis after drinking alcohol.

### Imaging and pathological findings

The imaging localization of most of the tumors was accurate, except for two lesions that were believed to originate from the liver or other surrounding tissues, with the largest diameters being 15 cm and 11 cm, respectively (8, 13). All tumors presented as well-defined masses with no obvious signs of surrounding invasion. Among 13 cases with more comprehensive imaging information (**Table 1**), five were pathologically proven to be solid, and all of them presented a heterogeneous texture (including descriptions such as heterogeneous, mixed-density, and uneven density). After contrast administration, tumors showed different degrees and patterns of enhancement. Of the six cases describing the degree of enhancement, three cases with moderate to marked enhancement were found to be tumors containing solid components pathologically, while two cases with mild or slight enhancement were confirmed to be cystic tumors. In terms of pattern, peripheral enhancement was observed in two cases, of which one was solid and one was cystic. Progressive enhancement and delayed washout were shown in one solid tumor. The 33 tumors ranged from 1.2 cm to 15.0 cm (mean, 5.2 cm; median, 4.0 cm) in greatest dimension. On the gross examination of these 33 tumor specimens, 19 were solid (median diameter, 3.5 cm), eight were solid-cystic (median diameter, 4.0 cm), and six were cystic (median diameter, 8.5 cm). Cystic components could be located in both peripheral and central parts of the tumors, varying in size and number, or filling the tumor with a spongy appearance. MR chemical shift imaging showed little change in signal intensity between in-phase and out-of-phase images. Two patients underwent positron-emission tomographic (PET) examinations, which showed high metabolic uptake of the tumors, with Standard Uptake Value (SUVs) of 3.4 and 4.6, respectively. Histology and imaging revealed calcification in a total of seven cases, with the tumor’s greatest diameter ranging from 2.5 cm to 15 cm (mean, 6.3 cm; median, 3.4 cm). Preoperative imaging showed three of them, which were later confirmed by gross examination. Four cases were found only in postoperative specimens, with most being microscopic (three of four), and thus, were not reflected on imaging. Hemorrhages occurred in five cases and tended to occur in tumors with larger diameters (median, 5.0; mean, 6.4). Necrosis was rarely observed in tumors.

### Preoperative diagnosis and prognosis

Of the 20 patients with available initial diagnosis, 11 were considered benign, including six adenomas, one cyst, one echinococcosis, one myelolipoma, one schwannoma or pheochromocytoma, and one ganglioneuroma. Six cases were suspected to be malignant, including two metastases, two lymphomas, one cortical adenocarcinoma, and one unspecified malignancy. The preoperative diagnosis for the remaining three cases was vague. No cases of local recurrence or metastatic disease have ever been reported in 16 patients during 8–177 months of follow-up.

## Discussion

ATs are benign neoplasms of mesothelial origin that often occur in the reproductive tracts. Primary ATs in the adrenal glands are extremely rare, and the majority of cases affect men. Contrary to some previous reports, our review found that the right adrenal gland appears to be more commonly involved than the left, consistent with the findings of Guan et al. (24) in 2021.

Since patients with adrenal ATs are usually asymptomatic, imaging techniques are particularly important for the detection of these masses. Although previous literature believed that the imaging findings of adrenal ATs were not specific, there was no relevant literature to prove it. Therefore, including our cases, we collected data on 33 adrenal AT patients and extracted the imaging descriptions from the article to summarize some features.

In terms of location, almost all tumors appear as well-demarcated masses without any signs of surrounding invasion in the retroperitoneal space above the kidney. However, when the tumor is huge, it can be mistaken for originating in neighboring tissues such as the liver, lymph, or nerve tissue. Most tumors show heterogeneous texture, which can become more obvious after enhancement. Even solid tumors are mostly heterogeneous on imaging, possibly due to the presence of microscopic sacs. The enhancement degree of tumors varies from mild to marked and appears to be higher in tumors with more solid components than in those where cystic components are dominant. Patterns of progressive enhancement with delayed washout can occasionally be observed. MR imaging has great advantages in distinguishing cystic components, as they can be judged by the classic signal of liquid. Moreover, the probability of cyst occurrence increased as the tumor grew. It is difficult to summarize the distribution of cystic components in the tumor, as well as their number and shape, based on available information. The PET scan showed that the levels of ^18^F-FDG uptake were in the range of malignant adrenal lesions. This may result from the presence of numerous lymphoid follicles, which is a classic characteristic of adrenal ATs. Calcification is not very common but can occur in very small tumors and is detected sensitively by CT, while hemorrhage is only found in tumors above a certain volume. Little adipose tissue is contained in tumors so no changes can be seen in chemical shift imaging. Necrosis is rarely observed.

Adrenal ATs should be distinguished from more common diseases in this region. Benign diseases include adenoma, myelolipoma, pheochromocytoma, schwannoma, ganglioneuroma, hemangioma, and lymphangioma. Malignant diseases include cortical adenocarcinoma, lymphoma, and metastasis.

Adrenal adenomas (ACAs) are typically 1–3 cm lesions with uniform low attenuation (mean, 10 HU or less) on noncontrast CT as a result of their abundant neutral lipid content. Han et al. (26) showed that combining the minimum attenuation value (< 0 HU) with CT histogram analysis (negative pixels, ≥ 10%) on plain CT can improve the accuracy of diagnosis. Large size, calcifications, hemorrhage, or cystic appearance are rare findings in ACAs. Adrenal myelolipomas contain macroscopic fat and exhibit even lower attenuation than ACAs, with a reported mean attenuation of − 74 HU (27). Pheochromocytomas are catecholamine-secreting neuroendocrine tumors. Most of these tumors enhance aggressively but show inconsistent contrast material washout. A heterogeneous enhancing lesion with multiple high-signal-intensity pockets or cysts may be the most commonly observed pattern on T2-weighted images (28). Symptoms related to adrenergic excess and elevated serum and urinary metanephrine levels (catecholamine metabolites) combined with common imaging patterns can aid in making a more confident diagnosis. Schwannomas appear as heterogeneously enhancing, hypoattenuating masses due to their combination of Antoni A and B patterns. MR images may show marked variability depending on the degree of degenerative change (29). Moreover, a mass with decreased attenuation measuring < 40 HU on nonenhanced CT, gradual delayed enhancement, and a whorled appearance on T2-weighted images may suggest a ganglioneuroma (30). Adrenal hemangiomas are vasoformative neoplasms characterized by well-defined margins, phleboliths, and peripheral nodular discontinuous enhancement. Signal intensity on T1-weighted images depends on the presence of hemorrhage or necrosis within the tumor (31). When the tumor presents as mainly cystic or entirely cystic, it can be easily misdiagnosed as a lymphangioma. A hypoattenuating, thin-walled multicystic lesion without internal enhancement is most suggestive of a lymphangioma. The calcification patterns can vary (32).

Adrenal cortical carcinomas (ACCs) are usually steroid-productive tumors that are relatively large in size. Their imaging characteristics include nonfatty CT attenuation (greater than 10 HU on unenhanced CT), heterogeneous enhancement with a peripheral predominance, cystic changes or necrosis in central areas, and a probable presence of aggressive vascular invasion (33). Lymphomas are soft in texture and can grow along the lacuna, forming irregular masses without compressing surrounding tissue. Given the highly cellular nature of lymphomatous masses, restricted diffusion is commonly seen (34). Lymphoma tends to surround vessels rather than displace them, which can be distinguished from ACCs (35). The most common tumors that metastasize to the adrenal glands are carcinomas (lung, breast, and colon), malignant melanoma, and lymphoma. In a patient with a known malignancy, metastases should be considered unless a definitive diagnosis of a benign lesion can be made. However, the imaging appearance of most of them is nonspecific.

Nevertheless, imaging techniques cannot reliably distinguish adrenal ATs from other tumors. A definitive diagnosis requires histological and immunohistochemical evidence. Microscopically, the tumor is often composed of multiple tubular structures, microcystic, and cystic regions, and frequently forms fissured and mutually anastomotic cavities lined with flattened endothelioid cells or eosinophilic epithelioid cells (1). Lymphocyte infiltration and aggregates can often be observed in the stroma. The mesothelial origin of the adenomatoid tumor is proven and commonly accepted. However, the adrenal gland is not lined by mesothelium. Proposed theories include the presence of mesothelial inclusions as the cells of origin, or a histogenesis from primitive mesenchymal cells associated with the Müllerian tract (1). The glandular-like, sometimes signet-ring-like pattern will possibly raise suspicion of adenocarcinoma (6). The absence of significant atypia and the correct mesothelial immunoprofile can help avoid this misdiagnosis. Positive mesothelial markers for adenomatoid tumors include calretinin, D2-40, and WT-1 (1, 36, 37). When extragenital tissues test positive for these markers, mesothelioma should be suspected, although the adrenal area is rarely involved. The first that comes to mind is diffuse malignant mesothelioma (MM), which is the most common. Despite the rather characteristic morphologic aspect of adenomatoid tumors, a focal adenomatoid pattern does exist in 5% of the epithelioid subtypes of diffuse MM (38). However, this MM mostly occurs in the pleura and shows diffuse invasion of the underlying tissues, with obvious atypia, prominent nucleoli, and mitotic activity. Compared with diffuse MM, localized MM is solitary and circumscribed both macroscopically and radiologically (39). It is also usually pleural and less often peritoneal. None of the reported cases of localized MM, however, seem to resemble adenomatoid tumors morphologically. As a marker of malignancy in mesothelial proliferations, BAP1 loss/mutation can be seen in the two types of MM mentioned above. Another lesion in the mesothelial family that should be differentiated is the well-differentiated papillary mesothelial tumor (WDPMT), which often occurs in the peritoneum. It is characterized by a papillary growth pattern. The majority of WDPMTs are benign, with a low mitotic count and absence of atypia, expressing intact BAP1. Only a small fraction may have invasive foci (40).

Immunohistochemically, the tumor cells can also be positive for epithelial markers such as AE1/AE3, CAM5.2, and CK7. Negative staining was observed for CD34, CD31, HMB45, Melan-A, Actin, Desmin, Syn, and CgA (24).

Given the benign biological behavior of this tumor, for an accidentally discovered adrenal mass, if a typical adenomatoid pattern with benign nuclear morphology as well as sufficient evidence of mesothelial origin can be accessed by fine needle aspiration cytology or intraoperative biopsy, removal of the gland may not be necessary. In this condition, long-term follow-up and periodic radiographic examinations are essential. In addition, genetic tests are also necessary to completely rule out the presence or potential of malignant mesothelioma. However, due to the extremely low incidence of adenomatoid tumors in the adrenal region and that no cases were diagnosed before excision, the long-term prognosis for patients with retained tumors is uncertain. Therefore, surgical removal remains the first-line therapy.

## Conclusion

Through our review, we found some nonspecific imaging features of adrenal ATs. Both CT and MR imaging show well-circumscribed masses with heterogeneous interiors, which become more obvious after enhancement. The degree of enhancement ranges from mild to marked and appears to be higher in tumors with more solid components compared to cystic ones. Progressive enhancement with delayed washout can occasionally be observed. Cystic components can be more easily distinguished by MR imaging. The distribution and pattern of cystic components in tumors vary, but it is presumed that larger tumors are more likely to contain cystic components. Calcification is not so common but can occur in very small tumors. Malignant signs such as direct extension to surrounding tissues, regional lymph node metastasis, or aggressive vascular invasion are absent. However, the high metabolic behavior of the tumor on ^18^F-FDG scans may lead to a diagnosis of malignancy. More cases are needed to confirm these features. The final diagnosis of an adenomatoid tumor still depends on pathology and immunohistochemistry results. Genetic testing is necessary to rule out malignant mesothelioma. Surgical removal remains the first choice for treatment.

## Data Availability

The original contributions presented in the study are included in the article/**Supplementary Material**. Further inquiries can be directed to the corresponding author.
