# Supplementary material for: Case report: Imaging of adrenal adenomatoid tumors: reports of two cases and review of literature
Source: Front Oncol. 2024 Oct 4;14:1435143. doi: 10.3389/fonc.2024.1435143 (PMC11486722; doi:10.3389/fonc.2024.1435143)
Supplement: Supplementary file 1 [file Table1.pdf]

Supplementary material | Clinicoradiological features of adenomatoid tumors of the adrenal gland.

Author and citation	No.	Age/Sex/Side	Clinical findings, symptons and other diseases	Greatest dimension (cm)	Imaging modalities	Location	Margin	Density/Intensity(noncontrast)	Contrast	Other features of imaging	Preoperative diagnosis	Gross findings	Haemorrhage/Calcification/Necrosis/Others findings in pathology	Follow-up
Angeles-Angeles et.al(1)	1	34/M/R	IFA（AIDS）	3.0	/	Correct	/	/	/		/	Solid	None	Died
Gasque et.al(2)	2	28/M/R	IRF(acute cholecystitis)	9.0	MRI	Correct	*	mainly solid(homogeneous, isointense to the spleen), peripheral cystic areas	marked enhancement(solid)	Absence of intravoxel fatty and water elements	*	Solid & Cystic	Necrosis(G)	16 months
Kim et.al(3)	3	33/M/L	IRF(hypertention,Proteinuria)	1.7	CT	Correct	*	*	*		Adenoma	Solid	None	*
CHUNG-PARK et.al(4)	4	51/M/R	IRF(hypertension of primary aldosteronism)	3.0	CT	Correct	well-circumscribed	*	*		Adenoma(aldosterone producing)	Solid	None	*
Isotalo et.al(5)	5	37/M/L	IFS	3.1	/	Correct	/	/	/		Metastatic adenocarcinoma (frozen section)	Solid & Cystic	None	40 months
	6	31/M/R	IRF(asymptomatic)	3.2	*	Correct	*	*	*		Metastatic adeno carcinoma (FNA)	Solid	None	*
	7	31/M/unspecified	IRF(syncope)	3.5	*	Correct	*	*	*		Adrenal cortical tumor	Solid	None	50 months
	8	64/M/L	IFA	1.2	/	Correct	/	/	/		Lymphangioma	Solid	None	Died
	9	44/M/L	IRF(hypertension)	3.2	*	Correct	*	*	*		Lymphangioma	Solid	Calcification(Micro)	177 months
Denicol et.al(6)	10	42/M/L	IRF(Renal colic,hypertension,left renal stones)	10.5	CT	Correct	*	heterogeneous	peripheral enhancement,hypodense in interior		*	Solid	*	3 years
Garg et.al(7)	11	46/M/R	Not mentioned	11.0	CT	Uncertain(hepatic,renal,or adrenal origin)	*	a cyst of hepatic, renal, or adrenal origin	*		Cyst(of hepatic, renal, or adrenal origin)	Cystic(multilocular,thinken wall)	Haemorrhage and Calcification(G)	*
	12	33/M/R	Not mentioned	4.2	CT、MRI	Correct	a mass without invasion into the surrounding tissue	*	*		*	Solid	*	1 year

Author and citation	No.	Age/Sex/Side	Clinical findings, symptons and other diseases	Greatest dimension (cm)	Imaging modalities	Location	Margin	Density/Intensity(noncontrast)	Contrast	Other features of imaging	Preoperative diagnosis	Gross findings	Haemorrhage/Calcification/Necrosis/Others findings in pathology	Follow-up
Hamamatsu et.al(8)	13	30/M/L	IFA(illness after drinking alcohol)	3.0	/	Correct	/	/	/		/	Solid	Calcification(Micro)	Died
Varkarakis et.al(9)	14	54/M/R	IRF(acute right flank pain,right renal stones)	3.6	CT、MRI	Correct	*	*	*	Calcified components	*	Solid	Heterotopic ossification	1 year
Fan et.al(10)	15	42/M/L	IRF(hypertension,left renal stones,right renal cyts)	2.5	CT	Correct	*	*	*	Calcified spots	Inactive adrenal tumour	Solid & Cystic(tiny)	Calcification(G)	*
Timonera et.al(11)	16	47/M/R	IRF(diverticulitis)	7.0	MRI	Correct	*	*	*	Lipid-poor	Adenoma(lipid-poor)	Solid	*	*
	17	52/M/R	IRF(hypertension)	5.5	CT、MRI	Correct	*	*	*		*	Solid & Cystic	Haemorrhage(G)	*
Hoffmann et.al(12)	18	26/M/R	IRF(asymptomatic)	15.0	CT	Incorrect(hepatic origin)	*	a giant cystic of the liver	*	Calcifications	Echinococcus cyst of the liver	Cystic(passed through by fibrous trabeculae)	*	*
Biscegli a et.al(13)	19	39/M/R	IRF(asymptomatic，cancer of the left colon 4 years ago)	5.5	CT	Correct	well-defined	hypodense	*		Adenoma(non-functioning)	Cystic(focal mural thickening and short endoluminal papillations)	*	*
El-Daly et.al(14)	20	51/M/L	IRF(asymptomatic)	*	CT、MRI	Correct	well-defined	solid	*		*	Solid & Cystic	Calcification(Micro)	*
Phitayakorn et.al(15)	21	22/M/R	IRF(mediastinal lymphadenopathy,HIV)	2.5	MRI、PET	Correct	*	*	atypical enhancement	SUV=3.4	Adenoma(non-functioning) /Malignancy	Solid	None	*
Limbach et.al(16)	22	24/M/L	IRF(SDHD mutation)	3.6	CT、MRI	Correct	*	heterogeneous	*		*	Solid	Haemorrhage(G)	*
Li et.al(17)	23	32/M/L	IRF(asymptomatic)	4.0	CT	Correct	smooth	uneven density	majority unenhancement, mild to moderate enhancement of a small part		*	Solid	Haemorrhage(G)	2 and a half years
Zhao et.al(18)	24	62/M/R	IRF(hypertension)	3.0	CT	Correct	well-demarcated	hypodense	slight peripheral enhancement		Adenoma(non-functional)	Cystic(tiny,thin-walled,spongy)	None	8 months

Author and citation	No.	Age/Sex/Side	Clinical findings, symptons and other diseases	Greatest dimension (cm)	Imaging modalities	Location	Margin	Density/Intensity(noncontrast)	Contrast	Other features of imaging	Preoperative diagnosis	Gross findings	Haemorrhage/Calcification/Necrosis/Others findings in pathology	Follow-up
Babinska et.al(19)	25	40/F/R	IRF(asymptomatic)	9.0	CT	Correct	well-circumscribed	*	*		Adrenal carcinoma(non-functional)	Solid	*	*
Sağlıcan et.al(20)	26	40/M/R	IRF(asymptomatic)	5.5	MRI	Correct	well-marginated	mostly hyperintense,internal hypointense(nodular and thin septal components) on STIR	enhancement of internal components	No signal changes between in phase and out of phase T1-weighted images	*	Solid & Cystic(spongy)	*	1 year
Krstevska et.al(21)	27	30/F/R	IRF(asymptomatic)	8.0	CT	Correct	well-demarcated	heterogenous,hypodense	*		Myelolipoma	Cystic(smooth inner surface,filled with gelatinous)	Haemorrhage(G)	4 years
Dietz et.al(22)	28	28/M/R	IRF(Chronic abdominal pain)	4.5	CT、PET	Correct	well-defined	mainly solid(heterogeneous),cystic component(hypodense),intermediate density zone		Concordant uptake of the solid and cystic areas(SUVmax =4.64)	Malignancy	Solid	None	*
Guan et.al(23)	29	30/M/R	IRF(palpitation and dizziness)	3.5	CT	Correct	well-demarcated	*	*		*	Solid	None	21 months
	30	31/M/L	IRF(asymptomatic)	8.0	CT	Correct	*	*	*		Adenoma	Solid & Cystic(tiny,thin wall)	None	8 months
Qi et.al(24)	31	50/M/R	IRF(asymptomatic)	9.0	CT	Correct	*	mixed-density,polycystic(uneven thickness of the cyst wall)	mild enhancement		*	Cystic(multilocular)	*	6 years
our cases	32	33/M/R	IRF(elevated CA125)	4.0	MRI	Correct	well-marginated	mainly cystic,peripheral solid(hyperintense on SPAIR)	heterogeneous marked enhancement(solid)		Schwannoma/Pheochromocytoma	Solid & Cystic(multiple)	None	43 months
	33	28/M/R	IRF(asymptomatic)	4.0	CT、MRI	Correct	well-defined	mixed-density,mainly solid(hypointense on T1 and hyperintense on T2)	moderate enhancement, impregnated progressively from the periphery to the center,delayed washout	No signal changes between in phase and out of phase images	Ganglioneuroma	Solid	None	22 months

*M, male; F, female, R, right adrenal gland; L, left adrenal gland; IRF, incidental radiographic finding; IFA, incidental finding during autopsy; IFS, incidental finding during surgery for unrelated reasons; FNA, fine needle aspiration; *, not mentioned in the article; /, did not do any imaging examination; &, and; G, observed in gross examination; Micro, observed only by microscopy. The greatest diameter of the tumor is recorded based on the resected specimen, and if not mentioned, the diameter measured on the imaging is substituted.*

References

1. Angeles-Angeles A, Reyes E, Munoz-Fernandez L, Angritt P. Adenomatoid Tumor of the Right Adrenal Gland in a Patient with AIDS. Endocr Pathol. 1997;8(1):59-64.
2. Rodrigo Gasque C, Martí-Bonmatí L, Dosdá R, Gonzalez Martinez A. MR imaging of a case of adenomatoid tumor of the adrenal gland. Eur Radiol. 1999;9(3):552-4.
3. Kim MJ, Ro JY. Pathologic quiz case: a 33-year-old man with an incidentally found left adrenal mass during workup for hypertension. Adenomatoid tumor of adrenal gland. Arch Pathol Lab Med. 2003;127(12):1633-4.
4. Chung-Park M, Yang JT, McHenry CR, Khiyami A. Adenomatoid tumor of the adrenal gland with micronodular adrenal cortical hyperplasia. Hum Pathol. 2003;34(8):818-21.
5. Isotalo PA, Keeney GL, Sebo TJ, Riehle DL, Cheville JC. Adenomatoid tumor of the adrenal gland: a clinicopathologic study of five cases and review of the literature. Am J Surg Pathol. 2003;27(7):969-77.
6. Denicol NT, Lemos FR, Koff WJ. Adenomatoid tumor of supra-renal gland. Int Braz J Urol. 2004;30(4):313-5.
7. Garg K, Lee P, Ro JY, Qu Z, Troncoso P, Ayala AG. Adenomatoid tumor of the adrenal gland: a clinicopathologic study of 3 cases. Ann Diagn Pathol. 2005;9(1):11-5.
8. Hamamatsu A, Arai T, Iwamoto M, Kato T, Sawabe M. Adenomatoid tumor of the adrenal gland: case report with immunohistochemical study. Pathol Int. 2005;55(10):665-9.
9. Varkarakis IM, Mufarrij P, Studeman KD, Jarrett TW. Adenomatoid of the adrenal gland. Urology. 2005;65(1):175.
10. Fan SQ, Jiang Y, Li D, Wei QY. Adenomatoid tumour of the left adrenal gland with concurrent left nephrolithiasis and left kidney cyst. Pathology. 2005;37(5):398-400.
11. Timonera ER, Paiva ME, Lopes JM, Eloy C, van der Kwast T, Asa SL. Composite adenomatoid tumor and myelolipoma of adrenal gland: report of 2 cases. Arch Pathol Lab Med. 2008;132(2):265-7.
12. Hoffmann M, Yedibela S, Dimmler A, Hohenberger W, Meyer T. Adenomatoid tumor of the adrenal gland mimicking an echinococcus cyst of the liver--a case report. Int J Surg. 2008;6(6):485-7.
13. Bisceglia M, Carosi I, Scillitani A, Pasquinelli G. Cystic lymphangioma-like adenomatoid tumor of the adrenal gland: Case presentation and review of the literature. Adv Anat Pathol. 2009;16(6):424-32.
14. El-Daly H, Rao P, Palazzo F, Gudi M. A rare entity of an unusual site: adenomatoid tumour of the adrenal gland: a case report and review of the literature. Patholog Res Int. 2010;2010:702472.
15. Phitayakorn R, Maclennan G, Sadow P, Wilhelm S. Adrenal adenomatoid tumor in a patient with human immunodeficiency virus. Rare Tumors. 2011;3(2):e21.
16. Limbach AL, Ni Y, Huang J, Eng C, Magi-Galluzzi C. Adenomatoid tumour of the adrenal gland in a patient with germline SDHD mutation: a case report and review of the literature. Pathology. 2011;43(5):495-8.
17. Li S, Wang X, Zhang S. Adenomatoid tumor of adrenal gland: a rare case report. Indian J Pathol Microbiol. 2013;56(3):319-21.
18. Zhao M, Li C, Zheng J, Yan M, Sun K, Wang Z. Cystic lymphangioma-like adenomatoid tumor of the adrenal gland: report of a rare case and review of the literature. Int J Clin Exp Pathol. 2013;6(5):943-50.
19. Babinska A, Peksa R, Swiątkowska-Stodulska R, Sworczak K. The collection of five interesting cases of adrenal tumors from one medical center. World J Surg Oncol. 2014;12:377.

20. Sağlıcan Y, Kurtulmus N, Tunca F, Süleyman E. Mesothelial derived adenomatoid tumour in a location devoid of mesothelium: adrenal adenomatoid tumour. BMJ Case Rep. 2015;2015.
21. Krstevska B, Mishevska SJ, Jovanovic R. Adenomatoid Tumor of the Adrenal Gland in Young Woman: From Clinical and Radiological to Pathological Study. Rare Tumors. 2016;8(4):6506.
22. Dietz M, Neyrand S, Dhomps A, Decaussin-Petrucci M, Tordo J. 18F-FDG PET/CT of a Rare Case of an Adenomatoid Tumor of the Adrenal Gland. Clin Nucl Med. 2020;45(7):e331-e3.
23. Guan J, Zhao C, Li H, Zhang W, Lin W, Tang L, et al. Adenomatoid Tumor of the Adrenal Gland: Report of Two Cases and Review of the Literature. Front Endocrinol (Lausanne). 2021;12:692553.
24. Qi HF, Chen LQ, Yang MQ, Li XF, Zhang HN, Zhang KX, et al. Primary adenomatoid tumor of the adrenal gland: A case report and literature review. Medicine (Baltimore). 2023;102(50):e36739.
